# What Is the Burden of Immunoglobulin Replacement Therapy in Adult Patients With Primary Immunodeficiencies? A Systematic Review

**DOI:** 10.3389/fimmu.2018.01308

**Published:** 2018-07-02

**Authors:** Georgina L. Jones, Katharina S. Vogt, Duncan Chambers, Mark Clowes, Anna Shrimpton

**Affiliations:** ^1^Department of Psychology, School of Social Sciences, Leeds Beckett University, Leeds, United Kingdom; ^2^School of Health and Related Research, University of Sheffield, Sheffield, United Kingdom; ^3^Clinical Immunology and Allergy Unit, Northern General Hospital, Sheffield Teaching Hospitals and NHS Foundation Trust, Sheffield, United Kingdom

**Keywords:** systematic review, primary immunodeficiencies, PID, immunoglobulin treatment, burden of treatment, health-related quality of life

## Abstract

**Background:**

Primary immunodeficiency disorders (PIDs) are a group of heterogeneous rare disorders, whereby the immune system is missing or not functioning adequately. For patients requiring treatment, the most common option is immunoglobulin replacement therapy (Ig). Treatment of PIDs is simultaneously associated with both improvements in health-related quality of life (HRQoL) and increased treatment burden.

**Objectives:**

This review sought to review studies investigating the burden of Ig treatment, synthesize evidence in relation to administration routes (subcutaneous or intravenous) and instruments used, as well as make recommendations for clinical and research applications in this area for patients aged 16 years and older.

**Methods:**

We searched Medline, EMBASE, and The Cochrane Library. Sifting of titles was performed by two reviewers, and the assessment of full-text articles by three. From a database which contained 3,770 unique results, 67 full texts were reviewed. Eventually, 17 studies were found to meet the inclusion criteria, and included in this review. Due to data heterogeneity, a narrative, descriptive synthesis of the evidence was undertaken.

**Results:**

Most studies were carried out in the USA/North America, used a prospective observational design and involved patients with common variable immune deficiency. Four studies measured the burden of receiving IVIg therapy and 13 measured SCIg therapy. A wide range of measures, primarily designed to measure aspects of treatment satisfaction (e.g., life quality index or a slightly modified version) and HRQoL (e.g., The Short Form-36) had been used.

**Conclusion:**

Lack of a parallel control group in most studies meant that changes in outcomes could be due to factors other than changes in the treatment regimen. However, overall, PID patients appeared to report little Ig treatment burden and were satisfied with either modality. However, patient preference appeared to be the delivery of the Ig treatment in the patient’s home and SCIg was preferred after switching from IVIg therapy. Individual differences appeared to affect treatment preference and therefore understanding the decision support needs of PID patients facing IG treatment choices would be valuable. Using a questionnaire specifically designed to measure the burden of Ig treatment from the patient’s perspective is recommended in future research.

## Introduction

Primary immunodeficiencies (PIDs) are a group of heterogeneous rare disorders whereby the immune system is missing or not working properly. Consequently, for people living with a PID this means that they have a reduced or absent natural defense against viruses, bacteria, or fungi and will be susceptible to frequent infections (1–3), which can have a profound negative impact upon their health-related quality of life (HRQoL) (2).

Immunoglobulin replacement therapy (Ig) is the only treatment for most PID patients. It involves undergoing a blood-based infusion at regular intervals to raise the antibodies needed to fight off infection. It can be administered either intravenously or subcutaneously. Although it is generally accepted that Ig treatment can dramatically improve HRQoL (2, 4), it may also be associated with a substantial treatment burden. Burden of treatment (BoT) is a concept which can be defined as the consequences of receiving treatment (these may be medication, therapies, or other interventions). It describes the “work of being a patient”—everything the patient needs to do to treat and manage their illness, for example, undergoing tests and investigations, visiting doctors, adhering to treatment regimens, and making lifestyle changes (5, 6).

Intravenous Ig treatment (IVIg) is typically administered in hospital and can be infused two, three, or four weekly lasting approximately 2–4 h per visit. The precise length of infusion will be dependent on dose and tolerance of the individual, although the three-weekly interval is most common. Subcutaneous Ig treatment (SCIg) is typically administered in the home but can be administered in the hospital depending on patients’ individual needs. Because it is harder for tissues to accept larger volumes of the product quickly *via* the SC route, it often means that patients will require more frequent infusions of smaller quantities, leading to an increased number of injections and localized reactions to needle injections (7, 8).

### Rationale

While there are numerous studies which have tried to measure the HRQoL of patients with a PID [e.g., Ref. (2, 3, 9, 10)], less attention has focused upon the burden of Ig treatment. BoT is an important concept because it may negatively affect adherence to treatment, HRQoL, disease management, and health care outcomes such as hospitalizations and survival (5). The outcomes of a 2015 systematic review reported that SCIg was associated with better treatment satisfaction compared to IVIg and therefore that switching trained patients with antibody deficiency from IVIg to SCIg may be advantageous (11).

## Objective and Research Question

The purpose of this systematic review was to (i) systematically identify studies that measured the burden of Ig treatments on the HRQoL of patients with primary immunodeficiencies, (ii) appraise and synthesize this evidence in relation to the different modes of Ig administration available and the instruments used and, and (iii) to make recommendations for future clinical and research applications in this area.

### Methods

#### Study Design

##### Inclusion Criteria

Included studies had to recruit adults (aged ≥ 16 years) receiving immunoglobulin replacement therapy for a PID and report a measure of the BoT from the patient’s perspective. Where both children and adults were recruited, studies were only included if data for adults could be extracted separately from data on children (or their parents and the samples were not mixed). Studies using HRQoL or treatment satisfaction questionnaires or qualitative assessments of treatment burden were eligible. Studies with comparative or non-comparative (e.g., before and after) designs were eligible. Studies were excluded if they reported data from the clinician or health service perspective only, were editorials, letters, systematic reviews, conference abstracts, or if they were published in languages other than English.

### Search Strategy: Data Sources

On 18th August 2015, we conducted searches on Medline (including Medline in Process), EMBASE, and The Cochrane Library. This was subsequently updated on 27th October 2017. Citations were imported into EndNote and duplicates deleted prior to scrutiny. The searching process aimed to identify studies which reported data on the burden of immunoglobulin therapy, from the patient’s perspective for primary immunodeficiencies.

The search strategy was developed by an information specialist (Mark Clowes) who undertook electronic searching to create a database of citations using the EndNote reference management system. The search process was recorded in detail with lists of databases searched, date search run, limits applied, number of hits, and duplication as per PRISMA guidelines (http://www.bmj.com/content/339/bmj.b2700).

The initial search consisted of terms relating to Ig therapy for PID. Due to the large number of different types of primary immunodeficiencies with over 250 being identified as of 2011 (12), it was not practical to search for every single type so instead we used a combination of umbrella terms for the major categories and specific terms for some of the most common types.

Further searches were conducted to identify studies measuring:

(i) the quality of life (QoL) of patients living with PID and
(ii) the BoT for those undergoing IGT.

QoL terms were based on a search filter devised by http://nicedsu.org.uk/wp-content/uploads/2016/03/TSD9-HSUV-values_FINAL.pdf.

BoT terms were based on previous studies: http://www.ncbi.nlm.nih.gov/pmc/articles/PMC3692487/bin/pmed.1001473.s007.doc and (6).

The unduplicated searches found a total of 4,002 results. After de-duplication of results from all the searches, the database contained 3,770 unique results. Results were imported in discrete sets with labels added to help identify which facet(s) of the topic they included. This accelerated the sifting process by allowing each set of results to be reviewed separately and in accordance with appropriate criteria for the topic of the article. No date limits were set on any of the searches. However, it was expected that most of the evidence would be recent. We did not limit results to English language studies but those in other languages were imported and screened separately. The MEDLINE search strategy is reported in Appendix A1 in Supplementary Material. Similar searches were conducted of EMBASE (and EMBASE Classic) and The Cochrane Library. Reference lists of relevant papers were also screened and citation searches run.

#### Study Selection and Data Extraction

Initial sifting of the search results was performed by one reviewer (Georgina L. Jones), and subsequently updated by Katharina S. Vogt. Assessment of full-text articles against the inclusion criteria was undertaken by three reviewers (Duncan Chambers, Katharina S. Vogt, and Georgina L. Jones) and uncertainties were resolved by discussion and consensus. Data on study and patient characteristics, outcome measures, and study findings were extracted from included studies by three reviewers (Duncan Chambers, Katharina S. Vogt, and Georgina L. Jones). Data extraction forms were developed in advance and tested on a small sample of studies prior to the main data extraction process. No formal assessment of quality (risk of bias) of the included studies was conducted because of the wide range of included study types, most of which have no generally accepted tools for quality assessment.

#### Data Synthesis

As this was not an effectiveness review and due to the heterogeneity of the data, a narrative and descriptive synthesis of the evidence was planned, concentrating on the different modes (SC and IV) and settings (home and hospital/clinic) available for Ig therapy. A meta-analysis would have been conducted had the data allowed for this. We also sought to establish the different ways in which studies have characterized and measured the BoT from the patients’ perspective, including issues of HRQoL, preference, and financial burden.

## Results

### Prisma Flow Diagram

**Figure 1:**
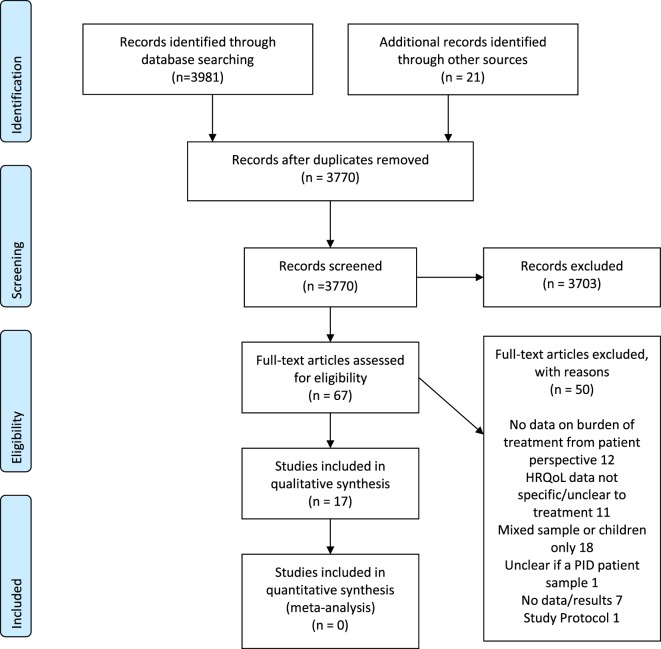
PRISMA 2009 flow diagram.

A PRISMA flow diagram is presented in Figure 1. Sixty-seven full texts were reviewed, of which 17 were included. Publication dates ranged between 1991 and 2017. Study characteristics are summarized in Table 1 and in more detail in Tables 2 and 3. Most studies were carried out in the USA/North America (*n* = 6), Sweden (*n* = 5), or other European countries (*n* = 2). The remainder involved patients from two or more countries (*n* = 4). Seven studies were prospective observational studies, mainly reporting changes from baseline (data collection ranged from 6 to 24 months), nine reported cross-sectional data (questionnaires administered at a single time point although one of these was a conjoint analysis study where patients expressed preferences for hypothetical treatment regimens with different features), and there was one crossover randomized trial.

**Table 1:** Summary table of study characteristics.

	Study design			Type of therapy			Measure of treatment burden		
Study	Prospective	Cross-sectional	Crossover	SCIg	IVIg	SCIg versus IVIg	QoL	Preference, satisfaction	Other
Chapel et al. (23)			√			√		√	
Daly et al. (17)		√			√		√	√	
Dash et al. (24)	√			√				√	
Gardulf et al. (26)	√			√				√	
Gardulf et al. (20)	√			√			√		
Gardulf et al. (14)		√		√				√	√
Gardulf et al. (25)		√		√					√ ($)
Gardulf et al. (30)		√		√					√
Hansen et al. (27)	√			√				√	
Howard et al. (15)		√			√		√		√ ($)
Jones et al. (13)	√			√			√	√	
Kittner et al. (28)		√				√		√	√
Mohamed et al. (19)		√ (conjoint analysis)				√		√	
Nicolay et al. (18)	√					√	√	√	
Rider et al (29),		√				√	√		√
Routes et al. (16)	√				√		√		
Tcheurekdjian et al. (22)		√			√		√		

*$: includes data on financial burden*.

*QoL, quality of life*.

**Table 2:** Study designs and outcome measures used.

Reference	Study design	Outcome measure
**IVIg general**		
Tcheurekdjian et al. (22)	Questionnaire (cross-sectional)	SF-36
Routes et al. (16)	Cohort study (12 months)	SF-36

**IVIg at home**		
None		

**SCIg at home**		
Dash et al. (24)	Cohort study (up to 4 years)	Satisfaction questionnaire
Gardulf et al. (26)	Prospective observational (12 months)	VAS (0–100)
Gardulf et al. (20)	Prospective observational (18 months)	67-item questionnaire developed for the study, SIP, and GHRI
Gardulf et al. (14)	Questionnaire (cross-sectional)	38-item questionnaire developed for the study, 8 items on perception of treatment
Gardulf et al. (30)	Questionnaire (cross-sectional)	VAS (0–100)
Hansen et al. (27)	Prospective observational (6 months)	VAS (0–100)
Jones et al. (13)	Prospective observational (up to 96 weeks)	SF-36, EQ-5D, LQI, TSQM, Ig therapy-specific questionnaire
Nicolay et al. (18)	Prospective observational (12 months)	SF-36, LQI

**Comparative studies**				
Chapel et al. (23)	Crossover randomized trial (24 months)	Not applicable (question about treatment preference)
Daly et al. (17)	Questionnaire (cross-sectional)	LQI, comparison scale for home versus clinic-based treatment, HOS, MHLC
Gardulf et al. (25)	Questionnaire (cross-sectional)	Cost questionnaire
Howard et al. (15)	Questionnaire (cross-sectional)	Questionnaire developed for the study, quality of life measured using SF-12
Kittner et al. (28)	Questionnaire (cross-sectional)	8-point Likert scales, 4 scales of FPI
Mohamed et al. (19)	Conjoint analysis	12-question conjoint survey offering choices between hypothetical treatments
Rider et al. (29)	Cohort study (cross-sectional)	SF-12, IDF survey

*EQ-5D, Euroqol-5D; FPI, Freiburg Personality Inventory; GHRI, General Health Rating Index; HOS, health opinion survey; IVIg, intravenous immunoglobulin; LQI, life quality index; MHLC, multi-dimensional health locus of control scale; SCIg, subcutaneous immunoglobulin; SF-36, short form-36; SF-12, short form 12; SIP, sickness impact profile; TSQM, treatment satisfaction questionnaire for medication; VAS, visual analogue scale*.

**Table 3:** Clinical characteristics of included studies.

Reference	Country	Type of PID	Ig treatment and place of administration	Brand and dose	Sample	*n*	Mean age and range
Chapel et al. (23)	UK and Sweden	CVID 18; IgG subclass deficiency 10; specific antibody deficiency 2	SCIg (setting unclear); IVIg (clinic-based)	SCIg: Gammabulin; IVIg: Endobulin (doses 400 mg/kg/month in UK, 600 mg/kg/month in Sweden)	Adults (>13 years), previously untreated or previously on prophylactic Ig therapy	30	44; range 18–67
Daly et al. (17)	USA	Not reported	IVIg (home- or hospital-based)	Sandoglobulin (dose not reported)	Participants in a trial of home-based IVIg and patients treated in a clinic setting at the same hospitals	37 home-based; 29 clinic-based	35.5 (home-based); 32.5 (clinic-based)
Dash et al. (24)	UK	CVID, IgGsubclasss deficiency, specific antibody deficiency, IGG heavy chain deficiency	SC (home)	Subgam (manufactured by BLP), initial weekly dose 100 mg/kg bodyweight—dose then individually adjusted	Adults and children enrolled in a trial of efficacy and safety of Subgam for home-infusion	28 adults	45.5 (21.3–75.2)
Gardulf et al. (26)	Sweden	Hypogammaglobulinaemia (further details not reported)	SCIg (home-based)	Gammaglobulin Kabi (100 mg/kg/week)	Consecutive patients receiving SCIg by rapid infusion at home	25	43; range 18–73
Gardulf et al. (20)	Sweden	CVID 23; XLA 1; other 1	SCIg (home-based)	Brand not reported (100 mg/kg/week)	Adults (≥18) with hypogammaglobulinaemia	25	43 (SD 16); range 18–66
Gardulf et al. (14)	Sweden; Denmark; and Norway	Not reported	SCIg (home-based)	Gammaglobulin Kabi (165 mg/ml); Gammabulin (160 mg/ml); and Nordimmun (150 mg/ml)	Adults receiving ongoing treatment with SCIg	152	44; range 18–76
Gardulf et al. (25)	Sweden	CVID or XLA (numbers not reported)	SCIg (home-based)	Brand not reported (100 mg/kg/week)	Adults who switched from hospital to home therapy	30	43; range 18–66
Gardulf et al. (30)	Sweden	cVID 6; IgG subclass deficiency 2; IgA and IgG2 subclass deficiency 1	SCIg (home-based)	Gammaglobulin or gammanorm (100 mg/kg/week)	Women who became pregnant while receiving SCIg at home	9	Mean not reported; range 25–43
Hansen et al. (27)	Sweden	IgG subclass deficiency 29; selective IgA deficiency 9; CVID 3; XLA 1; others 8	SCIg (home-based)	Gammanorm or immunoglobulin Baxter (100 mg/kg/week)	Patients who had been on rapid self-infusions at home for at least 6 months	50	Median 48; range 23–74
Howard et al. (15)	USA	XLA	IVIg (home- or hospital-based)	Not reported	Patients under care of the authors or who had participated in previous research studies	41	33; range 21–63
Jones et al. (13)	USA	CVID	SCIg (home-based)	Hizentra (dose not reported)	Patients from a previous study who agreed to enroll in an extension study	21 enrolled, 16 analyzed	47.2 (SD 14.5); range 22–69
Kittner et al. (28)	Germany	CVID 48; IgG subclass deficiency 1; hyper IgM syndrome 1; XLA 2; not stated 9	SCIg (home-based); IVIg (hospital-based)	Not reported	Patients who had switched to SCIg or opted to continue on IVIg	61 (33 SCIg and 28 IVIg)	SCIg 37 (SD 9.1); IVIg 51.2 (SD 14.5)
Mohamed et al. (19)	USA	CVID 219; XLA 8; other 25	SCIg or IVIg; home or doctor’s office, hospital, or clinic	Not reported	Patients recruited from the Immune Deficiency Foundation member panel	252	50.2 (SD 13.8); range 19–80
Nicolay et al. (18)	USA and Canada	CVID 34; XLA 10	SCIg (home-based)	Vivaglobin (160 mg/ml)	Participants in a longitudinal study who agreed to participate in a HRQoL sub-study. Patients had switched to home SCIg from hospital (group A) or home (group B) IVIg	44	Group A 36.1 (SD 13.6); group B 35.5 (SD 13.2)
Rider et al. (29)	USA	CVID, IgG subclass deficiency, agammaglobulinaemia, “other” PIDs	IV IG, SCIg, Im Ig (at home, infusion suite, hospital, or other)	Not reported	Adult patients registered on the US IDF patient contact database	945	Median: 52.9 (range 18–82)
Routes et al. (16)	UK; USA; and Brazil	CVID, agammaglobulinaemia, hypogammaglobulinaemia, specific antibody deficiency, and other	IVIg at home or hospital	Not reported	Adult and child patients receiving care *via* Jeffrey Modell Foundation Treatment Centres in the United States, Brazil, and the United Kingdom	21 adults	47.1 (24–67)
Tcheurekdjian et al. (22)	USA	CVID	IVIg (home-based and hospital-based)	Not reported	Adult (≥18) patients receiving IVIg therapy *via* University Hospitals of Cleveland	58	49.9 (SD 15.7); range 19.3–87.4

*CVID, common variable immune deficiency; IVIg; PID, primary immunodeficiency; SCIg; XLA, X-linked agammaglobulinaemi; HRQoL, health-related quality of life*.

Comparisons were made with the patients’ previous treatment and occasionally with a control group (not randomly selected). Some studies also compared patients’ HRQoL with that of the general population of their country (13) or patients with other health conditions. One study looked at the financial BoT on patients in Sweden but these data were published in 1995 (14). A US study from 2005 reported on patients’ problems in obtaining insurance and access to therapy (15).

### Study Selection and Study Characteristics

#### Patient and Treatment Demographics

Most of the patients in the studies had common variable immune deficiency (CVID). Other diagnoses included agammaglobulinaemia, IgG subclass deficiency, specific antibody deficiency, selective IgA deficiency, and hyper IgM syndromes. Sometimes the specific diagnosis was not included, only PID, or listed as “other.” Three studies did not include details of specific diagnoses. The majority of studies (*n* = 8) looked at BoT in relation to SCIg, the majority of which looked at SCIg treatment at home (*n* = 7). Four studies looked at IVIg, three were hospital treatment-only, and one compared home versus hospital treatment. Five studies compared SCIg versus IVIg. One study did not include details of treatment route or setting (16).

When the dose of Ig therapy is mentioned, this was at standard replacement dose of between 400 and 600 mg/kg/month. Some studies used a conversion factor of 1.37 when transferring patients from intravenous to subcutaneous treatment. Patients in these studies were receiving a variety of immunoglobulin brands. The brand used will have depended on local preference and availability at the time.

#### Measuring BoT

The studies used a wide range of measures to assess BoT (Table 1). Three studies used the life quality index (LQI) or a slightly modified version (13, 17, 18). This is a condition-specific instrument developed specifically to measure IVIg treatment satisfaction for patients with a PID. One study used the treatment satisfaction questionnaire for medication (TSQM) (13) which is a generic instrument that measures a patient’s treatment satisfaction. Other non-validated questionnaires or visual analog scales (VASs) developed specifically for the studies had also been used to measure BoT. Patient preferences for treatment had also been elicited using a conjoint approach in one study (19).

However, most studies (*n* = 8) used generic HRQoL questionnaires, the most common being the SF-36 or its short form version, the SF-12. The Sickness Impact Profile (SIP) General Health Rating Index (GHRI) (20), health opinion survey (HOS), and multi-dimensional health locus of control (MHLC) (17, 18, 21) were also adopted. The preference-based EQ-5D was only used in one study (13). Financial BoT was evaluated in two studies (15). The table below (Table 1) maps some of the key features of the included studies.

#### What Is the Burden of IVIg Therapy?

Four studies included patients only receiving IVIg therapy (15–17, 22).

#### IVIg Therapy: Home Versus Hospital

Four studies compared patients receiving IVIg at home versus in a hospital setting (15–17, 22). One study reported that patients receiving home-based treatment (*n* = 37) had higher LQI scores than clinic-based patients. They reported greater convenience, comfort, independence to travel, treatment schedule flexibility, and a more pleasant treatment atmosphere. They also reported less disruption of daily activities as well as reduced waiting time and treatment-associated travel and cost. Patients who subsequently returned to clinic-based therapy had higher LQI scores during home-based treatment. No group-differences were found for HOS and MHLC scores. However, the type of PIDs was not reported (17).

One study assessed the financial BoT in 41 US-patients with X-linked agammaglobulinemia (15). Overall, patients perceived the IV gammaglobulin treatment as burdensome and not the condition itself or its symptoms. Reasons for this were the difficulty with scheduling therapy appointments affecting their ability to travel. However, specific numbers were not reported and they did not distinguish between the two different settings where treatment was undertaken. They further reported that almost half had experienced difficulty obtaining or maintaining insurance, experiences included insurance or coverage denial, conditional exclusions, treatment delays, insurance cancelations, or reaching their lifetime cap. Finance-related consequences were not receiving the care they needed; one participant accumulated a $120,000 hospital bill. Excluding this participant, meant out-of-pocket expense for health care was $1,388 (median $500). While it was not the aim of the study to directly assess financial BoT between the settings, it was reported in terms of QoL impact: Using the SF-12 as a QoL measure, the authors found that treatment setting did not significantly affect HRQoL (15).

Using the SF-12’s extended version, the SF-36, Tcheurekdjian et al. (22) examined differences between IVIg treatment for CVID patients receiving the treatment at home or the clinic. While this study did not report their results separately, they did report that female gender and older age were associated with lower QoL but there was no effect of other variables (including treatment setting) on SF-36 outcomes.

While the heterogeneity makes a comparison of results difficult; overall, it appears as though home-based IVIg was preferable to receiving clinic-based treatment, but that treatment setting had no significant impact upon HRQoL outcomes.

Routes et al. (16) investigated HRQoL in patients with PID before and after having been on IVIg treatment for 12 months. The majority of the sample (88%) received IVIg at a hospital rather than at home (12%). As above, HRQoL was assessed using the SF-12. Significant improvements on patients’ ratings were found for physical health, general health, and social functioning. While it was also reported that patients had significantly less emergency room visits associated with their IG treatment, a number of patients (*n* = 12) reported an increase in days missed from work or school after starting their Ig treatment. However, this increase was not statistically significant.

#### SCIg Therapy

Thirteen studies measured the burden of receiving SC Ig therapy (13, 18–20, 23–29). However, none of the included studies focused exclusively on hospital or clinic-based therapy.

Gardulf et al. (25) specifically developed a 38-item questionnaire with eight items on patients’ perception of SCIg treatment; four of which concerned patients’ experiences of receiving SCIg in general (home and hospital). In a sample of Gothenburg patients who had spent a median time of 3 years on treatment, the majority were highly satisfied with subcutaneous treatment, perceived it as effective, and wished to continue. The authors also report three observations. First, older patients perceived the SG method as more comfortable, but were more anxious to retain the treatment. Second, patients who had received a greater number of infusions, had lower scores for comfort, and third, women were generally more positive relative to men. Out of the 152 patients, 112 patients had experience of intramuscular and/or intravenous Ig treatment. Participants were asked to rank the treatment routes in order of preference. One-hundred-and-four patients subsequently responded; 96 ranked SC first, three ranked the intramuscular method as their chosen preference and five ranked IV first. The answers to the open-ended questions were divided into two categories, positive and negative statements which were further divided into sub-themes. Positive answers to the open-ended questions were categorized as: a simple and easy method (*n* = 25), effective in preventing infections (*n* = 23), reduced or no local pain (*n* = 18), and no adverse systemic reactions (*n* = 10). Negative statements were categorized as: time consuming (*n* = 7), dependent on treatment (*n* = 6), clumsy method/much equipment needed (*n* = 4), local pain/worry in inserting needles (*n* = 4), local SC tissue reactions (*n* = 4), and ineffective in preventing infection (*n* = 3).

#### SCIg Therapy at Home

Six of these studies, and thus the majority of them, evaluated SC therapy at home only (13, 14, 20, 25–27, 30).

Overall, patients reported a positive experience of receiving SCIg at home. In order to assess patients’ perceptions of using SC therapy at home, three studies (26, 27, 30) used VASs. Gardulf et al. (26) reported that the mean VAS scores for “how do you find the subcutaneous treatment?” were 86 (range 50–99) at the start of home treatment and 84 (28–100) after 12 months. For “how determined are you to continue with treatment?” scores were 91 (60–100) and 94 (75–100), respectively. For corresponding questions about home treatment, mean scores were 94 (65–100) and 96 (85–100), respectively. There were no significant changes over time or requests to change the type or place of treatment.

Gardulf et al. (30), in a study which focused on pregnant women and Ig home treatment, found that six of the nine women reported no worry about receiving SCIg therapy during pregnancy. The other three estimated their worry in early pregnancy at 100, 33, and 9 on the VAS scale. Reasons included that the baby would feel the needle, how they would receive infusions when these could not be given in the abdominal wall, and that the dose would not be enough to prevent infection. None of the women were worried about receiving treatment at home during pregnancy.

Hansen et al.’s (27) patients perceived local tissue reactions to express SCIg infusion (35 ml/h) as less intense or unchanged compared with rapid infusion (20 ml/h). Median VAS score for local reactions was 16. Patients were positive about the home therapy regimen (median VAS at 6 months 96, range 72–100) and were keen to continue with express infusions (median VAS 98, range 73–100). Patients reported that the express regimen had made it easier for them to find time for the therapy (median VAS 95, range 64–100).

Gardulf et al. (20, 25) used specifically developed scales in their studies, among other measures. First, Gardulf et al. (20) used three measures in their study: a 67-item questionnaire, using a VAS format that was specifically developed for the study ranging from 1 to 100 mm whereby 100 indicated the biggest burden/problem, the SIP and the GHRI. Over the 18 months from baseline to follow-up, fears of infections and anxiety about the future decreased significantly. Patients also reported a significantly increased ability to participate in recreational activities. For the SIP, it was found that patients had significantly poorer functional status (versus a Swedish reference group) for total SIP score and subscales mobility, sleep and rest, household management, work, and recreation or pastimes. After 18 months, however, differences were only significant for ambulation, mobility, and social interaction. For the GHRI, a significant improvement in total and current health ratings were found at 18 months compared with baseline.

Using the remaining four items which specifically asked patients about SCIg home therapy, Gardulf et al. (25) found that their sample of 115 patients (median time on treatment 2 years 7 months) from Denmark, Sweden, and Norway receiving SCIg at home were overall highly satisfied with the treatment and wished to continue. Men scored higher than women for perceived control of timing of infusions. Identical to the division outlined above, answers to the open-ended questions regarding home treatment were classified as positive and negative. The positive statements were further categorized as independence/freedom/flexibility (*n* = 57), no travel to hospital (*n* = 14), less time off work or school (*n* = 4), a sense of being less sick or disabled (*n* = 3), and hospital resources freed for others (*n* = 3). Stated negatives, or disadvantages, were time consuming (*n* = 23), difficulties in storing materials (*n* = 5), and no skilled help available if needed (*n* = 3).

Jones et al. (13) used a combination of the SF-36, EQ-5D, LQI, treatment satisfaction questionnaire for medication (TSQM), and an Ig Therapy-Specific Questionnaire. The SF-36 and EQ-5D were administered at weeks 1, 24, 48, and 72 and the other measures at weeks 1 and 60. It was found that patients’ scores on the SF-36 scores did not differ significantly from US population norms except for general health, which was below the population norm. The EQ-5D Index Score was stable and within the 95% CI of the US population norm. LQI scores ranged from 77.3 to 92.3 across visits, indicating that the SCIg treatment had little impact upon daily activities. TSQM scores showed high satisfaction following treatment. Ig therapy-specific questionnaire scores reflected high satisfaction with current therapy and with receiving treatment at home.

#### SCIg: Home Versus Hospital Treatment

One study directly compared the cost associated with subcutaneous treatment at home versus at hospital (14). In this study, the treatment of 30 patients who were initially receiving hospital-based SG treatment was switched to home-based therapy. Patients completed cost-questionnaires during the initial hospital period before starting home therapy, and after 12 months of being on the home-treatment regime. It was found that switching to home therapy reduced the mean annual cost to the patients from SEK 22,360 to SEK 10,970 (US $2,865 to $1,405). Patients’ out-of-pocket expenses (excluding cost of time) fell from SEK 2,080 to SEK 310 ($265 to $40). Costs of attending the hospital for training varied widely between patients. However, overall the study concluded that home therapy significantly reduces the cost associated with SC treatment for patients.

Dash et al. (24) carried out a clinical effectiveness and safety trial with a subcutaneous Ig rapid infusion. However, they only measured treatment satisfaction and preference in their sample with a specifically developed questionnaire. Before the trial-began, the majority of the sample received IVIg, either at home or hospital (*n* = 22), compared to SCIg (*n* = 6). At evaluations of treatment satisfaction after 3 and 6-months post-treatment began with SCIg, the majority of patients preferred SCIg to their previous medication (89% and 76% at 3 and 6-months evaluations, respectively). The authors further report that 56% of their sample rated SCIg as more convenient than past treatment and 36% found it more comfortable.

#### IVIg Versus SCIg Therapy

Several studies aimed to compare Ig treatment burden in patients receiving IVIg or SCIg modalities or in those patients switching between these two treatment options.

Chapel et al. (23) investigated patients’ treatment preference in the context of an international multicentre study. Five of the UK patients preferred the IV route in comparison to four preferring SC. Out of the Swedish patients, 11 preferred IV compared to six indicating their preference for SC. Four patients had no preference. It is noteworthy that one patient declined to enter the SC phase because of preference for IV therapy and two patients declined to switch from SC to IV (preference and fear of virus transmission). The preference data include patients who withdrew.

The SF-36 and the LQI was used by Nicolay et al. (18), with patients who initially received IV treatment, in home or hospital, but were transferred to received SCIg treatment. Within the sample, patients were divided into two sub-groups: patients who previously received IV in a hospital setting (group A) and patients who received home treatment (group B). Patients in group A showed significant improvements on the SF-36 subscales for role-physical, vitality, and general health on all three LQI subscales and in satisfaction with route and place of treatment. Patients in group B only showed improvements for SF-36 general health. Most patients preferred SC administration (group A 81%, group B 69%) and treatment at home (group A 90%, group B 92%).

Finally, two studies contrasting IVIg with SCIg developed study-specific questionnaires (19, 28). Kittner et al.’s (28) study-specific questionnaire investigated patients’ attitudes toward home-based SCIg therapy, which asked them to answer on an 8-point Likert scale with values from 1 indicating “not at all” to 8 indicating “very much.” Four subscales of the Freiburg Personality Inventory (FPI) were also administered. Overall, IVIg treated patients, who did not want to change to SCIg treatment, were concerned about time required for self-administration (6.9 versus 3.6) and about severe adverse reactions at home (4.7 versus 1.7). On the item “I dislike to puncture myself,” included in the study-specific questionnaire, IVIg patients agreed more strongly than SC patients (5.3 versus 2.0). Generally, patients on SCIg appreciated their treatment (7.2), and cited increased flexibility (50%) as the main advantage. Interestingly, the FPI values were lower for SCIg patients for “physical complaints” and “emotional lability.”

Mohamed et al. (19) developed a 12-question conjoint survey offering choices between hypothetical treatments. It was found that patients preferred home setting, monthly frequency, fewer needle sticks, and shorter treatment duration to alternative choices. Interestingly, the mode of administration was the least important attribute but patients slightly preferred self-administration to administration by a health professional. The data for parents of children with PID were reported separately and are not reported in this review.

Rider et al. (29) used the SF-12 and a study-specific 75-item questionnaire to assess the HRQoL in adult patients with PIDs. Out of their sample (*n* = 945), 55% received IV and 45% received SC treatment. One question in the study-specific questionnaire assessed the effects of treatment on fatigue, it was found that a greater percentage of IV IG patients (46%) reported always feeling fatigued or low in energy compared to SC patients (29%). In addition, a greater amount of SC patients reported “never” to experiencing periods of fatigue or low energy compared to IV patients (31 versus 16%). Differences in scores on the SF-12 indicate “modestly” improved scores for mental health for SC IG patients compared to IV IG patients. Interestingly though, there were no differences in perceived BoT between the administration groups—accessed *via* the study-specific questionnaire.

## Discussion

### Summary of Findings

The aim of this review was to synthesize the evidence in relation to the burden of Ig therapy as reported from the patient’s perspective, and to make recommendations for future clinical and research applications. Overall, PID patients reported little Ig treatment burden and were satisfied with either modality. However, patient preference appeared to be the delivery of the Ig treatment in the patient’s home and SCIg was preferred after switching from IVIg therapy.

While there was less evidence in relation to IVIg treatment, patients receiving this in a home setting were satisfied with their treatment and wished to continue. Better HRQoL and financial savings, despite some trouble getting insurance cover, are recorded to be factors influencing this preference. Similarly, most studies reported high patient satisfaction with SC therapy at home and most patients who switched to this regimen preferred it to their previous treatment (IV hospital–based therapy), although one study reported no significant differences in terms of preferences about the setting in which SC treatment is received. In most studies, switching to SC home therapy was associated with improvements in some or all measures of HRQoL. Patients were largely positive, satisfied, and perceived the therapy as effective, giving them greater independence as well as flexibility. Other benefits cited included increased flexibility, convenience, and self-confidence associated with self-management of the condition. Aspects perceived as burdensome after switching to SC therapy at home included that it was time consuming, challenging to store equipment, and there was a lack of skilled help if needed.

Improvements on HRQoL measures as well as health functioning measures were recorded, especially when patients changed their treatment to SCIg at home from either IV hospital or IV home treatment. From these results, it could be concluded that as a result of increasing HRQoL and, potentially decreasing patients’ financial burden, SCIg therapy may be the most cost-effective as well as preferred treatment option for PID patients. However, more studies investigating the financial burden of different treatment administration routes need to be conducted to fully support this claim.

Furthermore, it also important to take factors into account that have been reported occasionally in some studies: the importance of individual differences. The choice of which route to use for Ig administration depends upon personal preference of the patient, ease of intravenous access, dose of Ig required, tolerability of any previous Ig products, patient lifestyle, and it can be reviewed regularly and adjusted throughout the period of treatment as patient’s circumstances change (31). Sometimes choice of product may also be limited by specific country’s insurance and financial arrangements.

Across studies, it was reported that women and men may have different attitudes and anxieties toward treatment and treatment success. In addition, the age of patients may play an important role. Those characteristics, as well as some personality traits, as measured by Kittner et al. (28), may significantly influence people’s preference and attitudes toward treatment and findings like this could be utilized in developing tailored interventions to reduce anxieties of particular groups of PID patients who face Ig treatment decisions.

It was surprising to find that none of the included studies in this review were randomized-control trials, but instead had adopted cross-sectional designs, which suggests that the area of immunoglobulin research for patients with PID requires further gold-standard research, especially in connection to HRQoL measures and assessment of BoT. However, before this research can be conducted, it is important to take account of the following: across the 22 studies included in this review, ten different “health” questionnaires were used (LQI, TSQM, CHQ-50, EQ-5D, FPI, SF-36/SF-12, GHRI, SIP, and HOS) (note: this list excludes the use of the VAS and other study-specific measures). This vast heterogeneity of different measures, subsequently measuring different outcomes does not enable direct comparison across results. A new Ig BoT specific measure is currently in the early stages of development and validation, which may be a useful measure for standardizing the measurement of Ig treatment burden across future studies and as newer Ig therapies and modalities are developed (32). Two other measures which assess HRQoL have already been published and validated, yet were not utilized (33, 34).

While some studies have attempted to gain a more comprehensive insight into which factors and characteristics may contribute (e.g., age, gender, and personality factors) and why certain treatments may be preferred (e.g., flexibility, freedom to travel, frequency of treatment, etc.), the evidence is not yet conclusive. Furthermore, data included in this review spans three decades from 1987 to October 2017 (when updated). With advances in technology as well as shifts in the delivery of health care to person-centered care, it is possible that people’s understanding, demands on and readiness to adapt their treatment regimens may have changed. Some of the financial burden reported may also now be out-dated and not applicable anymore as funding rules change. Furthermore, many studies emphasizing the superiority of SC compared to IV were conducted in single centers, rather than across multi-sites. This may have further biased the results considering the lack of comparisons that have taken place. It is also important to note that with the expansion of rapid infusion (IVIg), the length of treatment per session will have decreased and may, therefore, not be more time consuming than SC.

### Limitations

There are several limitations of this systematic review. First, in regards to the inclusion criteria it was decided to focus on adult patients. The age cutoff used was 16 years and older because this is used in NHS ethics protocols to distinguish between adult and pediatric cohorts in the UK. Unfortunately, a few interesting papers which were originally thought to have met the inclusion criteria were not included in the review because they had classified the adult group as any patient aged 14 years and above and therefore this did not meet our inclusion criteria and/or did not report child and adult data separately (10, 21, 35–40). One study was excluded because after removing the child cohort data, the data for only one adult patient remained, which was deemed insufficient (41). However, inclusion or exclusion of the most “marginal” studies would be unlikely to affect our overall review findings. These are that any Ig therapy at home was positive including IVIg or SCIg but SCIg at home appeared to be associated with less treatment burden and was the most desired mode of treatment in patients who had experienced both treatment modalities.

The choice of which route to use for Ig administration usually depends upon personal preference of the patient, ease of intravenous access, dose of Ig required, tolerability of any previous Ig products, patient lifestyle, and it can be reviewed regularly and adjusted throughout the period of treatment as patient’s circumstances change. However, it is worth mentioning that one study found IVIg treatment to be associated with higher levels of anxiety and depression. For example Heath et al. (42) specifically investigated anxiety and depression in adults with primary immunodeficiencies, and how their experiences of mental health relate to their illnesses. They assessed depression as well as anxiety with the Hamilton-Depression (HAM-D) and Hamilton-Anxiety (HAM-A) questionnaires. Their participants included adults who had either received IV IG, either at home or hospital, and patients receiving home-SC. While this was not intended as a direct comparison of treatment burden for both groups, the authors report some relevant findings: in regards to depression, it was found that patients receiving IVIg (home or hospital) scored significantly higher on the HAM-D scale than SC patients, and thus were significantly more depressed than SC patients (*p* = 0.0004). The authors concluded that receiving IVIg treatment may, therefore, be a risk factor for PID patients for developing depression. For the HAM-A, the relevant finding to this review is that IVIg patients attributed higher percentages of their anxiety to their PID diagnoses compared to patients receiving SCIg (*p* = 0.030). Monitoring the psychological well-being of PID patients receiving IVIg therapy especially may, therefore, be needed although more research is warranted to investigate this possible relationship further.

The main limitation of the evidence identified from this review is the lack of direct comparisons between patients who have only ever received one modality of treatment, which means that changes in outcomes like HRQoL or treatment burden could be due to factors other than changes in the treatment regimen. In addition, many of the included studies were performed by a relatively small group of authors with small sample sizes and it is likely that some of the same patients were included in multiple studies which would have the effect of exaggerating the quantity of evidence available, and would bias the results of this review significantly.

## Conclusion and Recommendations for Future Research

This study presents a summary of the evidence concerning the perceived burden of Ig treatment from the patient’s perspective. Specifically, this review assessed relevant concepts, such as HRQoL, patient satisfaction, treatment preference, and subjective experiences of patients. These are all aspects relevant to the BoT, but potentially also the burden of disease.

Overall, it appeared that PID patients reported little Ig treatment burden. Ig therapy at home appears preferable to clinic-based treatment and SCIg at home was the most desirable treatment modality. However, individual differences do affect treatment preference and exploring and identifying the decision support needs of PID patients facing IG treatment choices would be valuable using a shared-decision making approach.

However, the heterogeneity of the disease specific and generic outcome measures used to assess treatment burden in these studies, none of which directly assess the BoT, and the use of non-validated measures makes synthesizing the current evidence concerning Ig treatment burden difficult. If HRQoL is the primary outcome of interest then a HRQoL instrument is most appropriate to use. However, if the intent is to measure BoT then such a measure should be selected. Indeed, a measure specifically designed to measure Ig treatment burden from the patient’s perspective may therefore be of value and one is currently in the early stages of development and testing (Jones et al., unpublished). Future research to explore how perceived adult IG treatment burden may differ to children’s perceptions of Ig treatment burden would be recommended—especially as patients need to stay on Ig treatment for the duration of their lives and this information could be used to better support patients transitioning between pediatric and adult centers for treatment.

## Author Contributions

GJ: conceived the idea, developed the protocol, undertook the sifting and appraisal of the papers, and co-wrote the paper for publication. KV: undertook the sifting and appraisal of the papers, co-wrote the paper for publication. DC: undertook the sifting and appraisal of the papers and supported drafting of the paper for publication. MC: provided the information resource expertise and undertook the searches of the literature. AS: provided clinical expertise and helped with drafting of the manuscript for publication.

## Conflict of Interest Statement

The authors declare that this study received funding from Shire. The funder was not involved in the study design or collection, analysis or interpretation of the data presented in this manuscript. This paper was prepared in the context of the International Patient Organisation for Primary Immunodeficiencies (IPOPI) BoT Study.
