# Supplementary material for: What Is the Burden of Immunoglobulin Replacement Therapy in Adult Patients With Primary Immunodeficiencies? A Systematic Review
Source: Front Immunol. 2018 Jul 2;9:1308. doi: 10.3389/fimmu.2018.01308 (PMC6043812; doi:10.3389/fimmu.2018.01308)
Supplement: Supplementary file 1 [file data_sheet_1.PDF]

Appendix1: MEDLINE search strategy

MEDLINE 1946 to 2015 (& Medline In Process) via Ovid Online (accessed 18th August 2015)

Search Strategy:

#	Searches
1	PID.mp. [mp=title, abstract, original title, name of substance word, subject heading word, keyword heading word, protocol supplementary concept word, rare disease supplementary concept word, unique identifier]
2	Primary immunodeficienc*.mp. [mp=title, abstract, original title, name of substance word, subject heading word, keyword heading word, protocol supplementary concept word, rare disease supplementary concept word, unique identifier]
3	Primary antibody deficienc*.mp. [mp=title, abstract, original title, name of substance word, subject heading word, keyword heading word, protocol supplementary concept word, rare disease supplementary concept word, unique identifier]
4	common variable immune deficienc*.mp. [mp=title, abstract, original title, name of substance word, subject heading word, keyword heading word, protocol supplementary concept word, rare disease supplementary concept word, unique identifier]
5	CVID.mp. [mp=title, abstract, original title, name of substance word, subject heading word, keyword heading word, protocol supplementary concept word, rare disease supplementary concept word, unique identifier]
6	x linked agammaglobulinaemia.mp. [mp=title, abstract, original title, name of substance word, subject heading word, keyword heading word, protocol supplementary concept word, rare disease supplementary concept word, unique identifier]
7	XLA.mp. [mp=title, abstract, original title, name of substance word, subject heading word, keyword heading word, protocol supplementary concept word, rare disease supplementary concept word, unique identifier]
8	(deficien* adj3 specific antibod*).mp. [mp=title, abstract, original title, name of substance word, subject heading word, keyword heading word, protocol supplementary concept word, rare disease supplementary concept word, unique identifier]

9	(primary adj3 antibod*).mp. [mp=title, abstract, original title, name of substance word, subject heading word, keyword heading word, protocol supplementary concept word, rare disease supplementary concept word, unique identifier]
10	(primary adj3 immune).mp. [mp=title, abstract, original title, name of substance word, subject heading word, keyword heading word, protocol supplementary concept word, rare disease supplementary concept word, unique identifier]
11	(primary adj3 immunodeficien*).mp. [mp=title, abstract, original title, name of substance word, subject heading word, keyword heading word, protocol supplementary concept word, rare disease supplementary concept word, unique identifier]
12	(antibod* adj3 deficien*).mp. [mp=title, abstract, original title, name of substance word, subject heading word, keyword heading word, protocol supplementary concept word, rare disease supplementary concept word, unique identifier]
13	ig* deficien*.mp. [mp=title, abstract, original title, name of substance word, subject heading word, keyword heading word, protocol supplementary concept word, rare disease supplementary concept word, unique identifier]
14	PID.mp. [mp=title, abstract, original title, name of substance word, subject heading word, keyword heading word, protocol supplementary concept word, rare disease supplementary concept word, unique identifier]
15	X-SCID.mp. [mp=title, abstract, original title, name of substance word, subject heading word, keyword heading word, protocol supplementary concept word, rare disease supplementary concept word, unique identifier]
16	Combined immunodeficiencies.mp. [mp=title, abstract, original title, name of substance word, subject heading word, keyword heading word, protocol supplementary concept word, rare disease supplementary concept word, unique identifier]
17	X-linked severe combined immunodeficiency.mp. [mp=title, abstract, original title, name of substance word, subject heading word, keyword heading word, protocol supplementary concept word, rare disease supplementary concept word, unique identifier]
18	(("adenosine deaminase" or ADA) adj2 deficien*).mp. [mp=title, abstract, original title, name of substance word, subject heading word, keyword heading word, protocol supplementary concept word, rare disease supplementary concept word, unique identifier]

19	Well-defined syndromes with immunodeficiency.mp. [mp=title, abstract, original title, name of substance word, subject heading word, keyword heading word, protocol supplementary concept word, rare disease supplementary concept word, unique identifier]
20	Wiskott Aldrich syndrome.mp. [mp=title, abstract, original title, name of substance word, subject heading word, keyword heading word, protocol supplementary concept word, rare disease supplementary concept word, unique identifier]
21	DiGeorge syndrome.mp. [mp=title, abstract, original title, name of substance word, subject heading word, keyword heading word, protocol supplementary concept word, rare disease supplementary concept word, unique identifier]
22	Di George syndrome.mp. [mp=title, abstract, original title, name of substance word, subject heading word, keyword heading word, protocol supplementary concept word, rare disease supplementary concept word, unique identifier]
23	Hyper-IgE syndrome*.mp. [mp=title, abstract, original title, name of substance word, subject heading word, keyword heading word, protocol supplementary concept word, rare disease supplementary concept word, unique identifier]
24	Predominantly antibody deficienc*.mp. [mp=title, abstract, original title, name of substance word, subject heading word, keyword heading word, protocol supplementary concept word, rare disease supplementary concept word, unique identifier]
25	common variable immune deficienc*.mp. [mp=title, abstract, original title, name of substance word, subject heading word, keyword heading word, protocol supplementary concept word, rare disease supplementary concept word, unique identifier]
26	CVID.mp. [mp=title, abstract, original title, name of substance word, subject heading word, keyword heading word, protocol supplementary concept word, rare disease supplementary concept word, unique identifier]
27	X-linked agammaglobulin?emia.mp. [mp=title, abstract, original title, name of substance word, subject heading word, keyword heading word, protocol supplementary concept word, rare disease supplementary concept word, unique identifier]
28	XLA.mp. [mp=title, abstract, original title, name of substance word, subject heading word, keyword heading word, protocol supplementary concept word, rare disease supplementary concept word, unique identifier]

29	Hyper IgM syndrome*.mp. [mp=title, abstract, original title, name of substance word, subject heading word, keyword heading word, protocol supplementary concept word, rare disease supplementary concept word, unique identifier]
30	IgG subclass deficienc*.mp. [mp=title, abstract, original title, name of substance word, subject heading word, keyword heading word, protocol supplementary concept word, rare disease supplementary concept word, unique identifier]
31	IgG sub-class deficienc*.mp. [mp=title, abstract, original title, name of substance word, subject heading word, keyword heading word, protocol supplementary concept word, rare disease supplementary concept word, unique identifier]
32	Transient hypogammaglobulin?emia.mp. [mp=title, abstract, original title, name of substance word, subject heading word, keyword heading word, protocol supplementary concept word, rare disease supplementary concept word, unique identifier]
33	Diseases of immune dysregulation.mp. [mp=title, abstract, original title, name of substance word, subject heading word, keyword heading word, protocol supplementary concept word, rare disease supplementary concept word, unique identifier]
34	immune dysregulation.mp. [mp=title, abstract, original title, name of substance word, subject heading word, keyword heading word, protocol supplementary concept word, rare disease supplementary concept word, unique identifier]
35	Chediak Higashi syndrome.mp. [mp=title, abstract, original title, name of substance word, subject heading word, keyword heading word, protocol supplementary concept word, rare disease supplementary concept word, unique identifier]
36	X linked lymphoproliferative syndrome.mp. [mp=title, abstract, original title, name of substance word, subject heading word, keyword heading word, protocol supplementary concept word, rare disease supplementary concept word, unique identifier]
37	X linked lympho-proliferative syndrome.mp. [mp=title, abstract, original title, name of substance word, subject heading word, keyword heading word, protocol supplementary concept word, rare disease supplementary concept word, unique identifier]
38	XLP.mp. [mp=title, abstract, original title, name of substance word, subject heading word, keyword heading word, protocol supplementary concept word, rare disease supplementary concept word, unique identifier]

39	Congenital defect* of phagocyte number.mp. [mp=title, abstract, original title, name of substance word, subject heading word, keyword heading word, protocol supplementary concept word, rare disease supplementary concept word, unique identifier]
40	Congenital defect* of phagocyte function.mp. [mp=title, abstract, original title, name of substance word, subject heading word, keyword heading word, protocol supplementary concept word, rare disease supplementary concept word, unique identifier]
41	chronic granulomatous disorder.mp. [mp=title, abstract, original title, name of substance word, subject heading word, keyword heading word, protocol supplementary concept word, rare disease supplementary concept word, unique identifier]
42	CGD.mp. [mp=title, abstract, original title, name of substance word, subject heading word, keyword heading word, protocol supplementary concept word, rare disease supplementary concept word, unique identifier]
43	barth syndrome.mp. [mp=title, abstract, original title, name of substance word, subject heading word, keyword heading word, protocol supplementary concept word, rare disease supplementary concept word, unique identifier]
44	defect* in innate immunity.mp. [mp=title, abstract, original title, name of substance word, subject heading word, keyword heading word, protocol supplementary concept word, rare disease supplementary concept word, unique identifier]
45	nemo deficiency.mp. [mp=title, abstract, original title, name of substance word, subject heading word, keyword heading word, protocol supplementary concept word, rare disease supplementary concept word, unique identifier]
46	Autoinflammatory disorder*.mp. [mp=title, abstract, original title, name of substance word, subject heading word, keyword heading word, protocol supplementary concept word, rare disease supplementary concept word, unique identifier]
47	Auto-inflammatory disorder*.mp. [mp=title, abstract, original title, name of substance word, subject heading word, keyword heading word, protocol supplementary concept word, rare disease supplementary concept word, unique identifier]
48	Muckle Wells syndrome.mp. [mp=title, abstract, original title, name of substance word, subject heading word, keyword heading word, protocol supplementary concept word, rare disease supplementary concept word, unique identifier]

49	Blau syndrome.mp. [mp=title, abstract, original title, name of substance word, subject heading word, keyword heading word, protocol supplementary concept word, rare disease supplementary concept word, unique identifier]
50	Complement deficienc*.mp. [mp=title, abstract, original title, name of substance word, subject heading word, keyword heading word, protocol supplementary concept word, rare disease supplementary concept word, unique identifier]
51	1 or 2 or 3 or 4 or 5 or 6 or 7 or 8 or 9 or 10 or 11 or 12 or 13 or 14 or 15 or 16 or 17 or 18 or 19 or 20 or 21 or 22 or 23 or 24 or 25 or 26 or 27 or 28 or 29 or 30 or 31 or 32 or 33 or 34 or 35 or 36 or 37 or 38 or 39 or 40 or 41 or 42 or 43 or 44 or 45 or 46 or 47 or 48 or 49 or 50
52	exp Immunoglobulins/ad, ae, ct, tu [Administration & Dosage, Adverse Effects, Contraindications, Therapeutic Use]
53	IVIg.mp. [mp=title, abstract, original title, name of substance word, subject heading word, keyword heading word, protocol supplementary concept word, rare disease supplementary concept word, unique identifier]
54	SCIg.mp. [mp=title, abstract, original title, name of substance word, subject heading word, keyword heading word, protocol supplementary concept word, rare disease supplementary concept word, unique identifier]
55	((immunoglobulin* or IG) adj3 (replac* or therap* or donation* or donor* or infus* or intraven* or intra-ven* or subcutane* or sub-cutane* or dose* or dosage* or infus* or administ* or treatment* or immunomodulat*)).mp.
56	52 or 53 or 54 or 55
57	health related quality of life.tw.
58	health related qol.tw.
59	health related ql.tw.
60	hrqol.tw.
61	hql.tw.
62	health state utilit$.tw.
63	hsuv$.tw.

64	(euroqol or euro qol or eq5d or eq 5d).tw.
65	(sf6d or sf 6d or sf 6 dimension$ or sf six dimension$ or shortform 6d or shortform six dimension$ or short form 6d or short form 6 dimension$ or short form six dimension$).tw.
66	(sf12 or sf 12 or short form 12 or shortform 12 or sf twelve or sftwelve or shortform twelve or short form twelve).tw.
67	(sf36 or sf 36 or short form 36 or shortform 36 or sf thirtysix or sf thirty six or shortform thirtysix or shortform thirty six or short form thirtysix or short form thirty six).tw.
68	(item adj3 short form).tw.
69	(item adj3 shortform).tw.
70	medical outcomes survey.tw.
71	medical outcomes study.tw.
72	mos.tw.
73	psychological general wellbeing index.tw.
74	psychological general well being index.tw.
75	pgwb$.tw.
76	health utilit$.tw.
77	hui$.tw.
78	quality of wellbeing.tw.
79	quality of well being.tw.
80	qwb$.tw.
81	rosser.tw.
82	trade off$.tw.
83	standard gamble$.tw.
84	tto$.tw.

85	qaly$.tw.
86	quality adjusted life year$.tw.
87	quality-adjusted life years/
88	hye$.tw.
89	health$ year$ equivalent$.tw.
90	disutilit$.tw.
91	disbenefit$.tw.
92	"Quality of Life"/
93	"Outcome Assessment (Health Care)"/
94	quality of life.tw.
95	92 or 93 or 94
96	(preference based or utilit$ or generic preference).tw.
97	95 and 96
98	(preference$ adj2 (elicit$ or patient$ or population$ or measure$ or based or cost$)).tw.
99	(utilit$ adj2 (elicit$ or patient$ or population$ or measure$ or based or cost$)).tw.
100	or/57-91
101	97 or 98 or 99 or 100
102	(burden* adj2 (treat* or therap*)).mp. [mp=title, abstract, original title, name of substance word, subject heading word, keyword heading word, protocol supplementary concept word, rare disease supplementary concept word, unique identifier]
103	activities of daily living/ or health behavior/ or health, knowledge, attitudes, practice/ or lifestyle/ or occupational therapy/ or palliat*.mp. or patient education as topic/ or exp rehabilitation/ or exp physical therapy modalities/ or self care/ or barthel*.mp. [mp=title, abstract, original title, name of substance word, subject heading word, keyword heading word,

	protocol supplementary concept word, rare disease supplementary concept word, unique identifier]
104	Disabled Persons/ or Disability Evaluation/ or work capacity evaluation/ or functional disability.mp.
105	social support/ or exp family/px or exercise/ or "physical education and training"/ or physical endurance/ or fatigue/ or physical fitness/ or long term care/
106	exp Gait/ or exp Gait Disorders, Neurologic/ or exp Accidental Falls/
107	self administration/ or (treatment adj3 (regimen* or regime* or tasks or work)).mp. or unnecessary*.mp. or unmet.mp.
108	exp cognition disorders/ or independen*.mp. or dependenc*.mp. or socioeconomic factors/ or life change events/
109	or/103-108
110	travel*.mp. or automobile driving/ or mobility limitation/ or transportation/
111	(recover* or challeng* or goal*).mp.
112	interpersonal relations/ or social isolation/ or cost of illness/ or schedul*.mp.
113	or/110-112
114	109 and 113
115	adaptation, psychological/ or adheren*.mp. or nonadheren*.mp. or patient compliance/ or noncomplian*.mp. or inconvenien*.mp. or negotiat*.mp. or (patient adj2 (care or experience or understand* or expectation* or perspective*)).mp. or patient satisfaction/ or personal autonomy/ or physician-patient relations/ or professional-patient relations/ or dissatis*.mp. or quality of life/
116	self concept/ or self care/ or self-management/ or suffer*.mp.
117	attitude/ or attitude to health/
118	115 or 116 or 117
119	109 and 118

120	109 and (exp leisure activities/ or health status/ or well-being.mp.)
121	109 and (Health Services Accessibility/ or continuity of patient care/ or disrupt*.mp.)
122	sickness impact profile/
123	needs assessment/ and burden*.mp.
124	102 or 114 or 119 or 120 or 121 or 122 or 123
125	51 and 56 and 101
126	51 and 56 and 124
127	51 and 101
128	56 and 124
129	125 or 126 or 127 or 128
